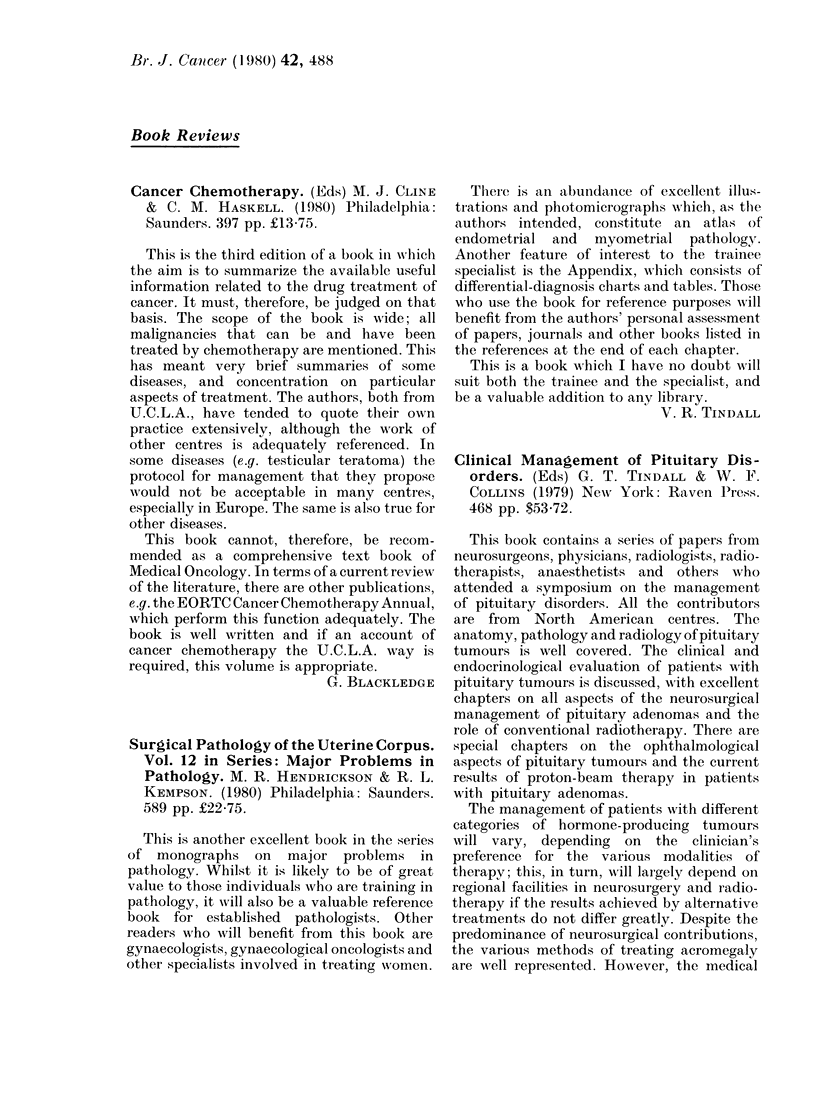# Surgical Pathology of the Uterine Corpus. Vol. 12 in Series: Major Problems in Pathology

**Published:** 1980-09

**Authors:** V. R. Tindall


**Surgical Pathology of the Uterine Corpus. Vol. 12 in Series: Major Problems in Pathology.** M. R. Hendrickson & R. L. Kempson. (1980) Philadelphia: Saunders. 589 pp. £22·75.

This is another excellent book in the series of monographs on major problems in pathology. Whilst it is likely to be of great value to those individuals who are training in pathology, it will also be a valuable reference book for established pathologists. Other readers who will benefit from this book are gynaecologists, gynaecological oncologists and other specialists involved in treating women.

There is an abundance of excellent illustrations and photomicrographs which, as the authors intended, constitute an atlas of endometrial and myometrial pathology. Another feature of interest to the trainee specialist is the Appendix, which consists of differential-diagnosis charts and tables. Those who use the book for reference purposes will benefit from the authors' personal assessment of papers, journals and other books listed in the references at the end of each chapter.

This is a book which I have no doubt will suit both the trainee and the specialist, and be a valuable addition to any library.

V. R. Tindall